# A Long-Term Stable Sensor Based on Fe@PCN-224 for Rapid and Quantitative Detection of H_2_O_2_ in Fishery Products

**DOI:** 10.3390/foods10020419

**Published:** 2021-02-14

**Authors:** Pei Hu, Zhentao Sun, Yunwen Shen, Yiwen Pan

**Affiliations:** Ocean College, Zhejiang University, Zhoushan 316021, China; hupei@zju.edu.cn (P.H.); szt@zju.edu.cn (Z.S.); 21934032@zju.edu.cn (Y.S.)

**Keywords:** Fe@PCN-224, Nafion, nonenzymatic sensor, hydrogen peroxide, fishery products

## Abstract

Hydrogen peroxide (H_2_O_2_) has been reported to be used for the illegal treatment of fishery products in order to obtain “fake” freshness. Residues of H_2_O_2_ in food may be of toxicology concern. In this study, a nonenzymatic sensor was developed based on Fe@PCN-224 metal–organic frameworks wrapped by Nafion to detect H_2_O_2_ concentration. The hybrid structure of Fe@PCN-224 was fabricated by incorporated free Fe^III^ ions into the center of PCN-224, which was ultra-stable due to the strong interactions between Zr_6_ and the carboxyl group. Scanning electron spectroscopy images exhibited that Nafion sheets crossed together on the surface of Fe@PCN-224 nanoparticles to form a hierarchical and coherent structure for efficient electron transfer. Electrochemical investigations showed that the Fe@PCN-224/Nafion/GCE possessed good linearity from 2 to 13,000 μM (including four orders of magnitude), low detection limits (0.7 μM), high stability in continuous monitoring (current remained nearly stable over 2300 s) and in long-term measurement (current decreased 3.4% for 30 days). The prepared nanohybrid modified electrode was effectively applied to H_2_O_2_ detection in three different fishery products. The results were comparable to those measured using photometrical methods. The developed electrochemical method has a great potential in detecting the illegal management of fishery products with H_2_O_2_.

## 1. Introduction

Nowadays, food quality control has become increasingly important due to the growing demand for high-quality and sanitary food [1,2]. Among a variety of foods, the freshness of fishery products is the most crucial commercial quality factor for consumers [3,4,5]. The Regulation (EC) N 2406/96 of the European Parliament and the Council defines four categories for fresh fish products. The fishery products classified as the last category must be judged as not suitable for humans consumption and withdrawn from the market [6].

Because of the actions of many endogenous and exogenous enzymes, fishery products are perishable and easy to have appearance changes with off-flavors [7]. However, some illegal treatments on these products may simulate “fake” freshness, one of which is to use hydrogen peroxide (H_2_O_2_). Illegal treatment with 0.5–0.8% H_2_O_2_ aqueous solution has been reported [8], which causes whitening and “fresh” effects on fishery products due to the oxidation properties of H_2_O_2_ [9,10].

In fact, H_2_O_2,_ with ready availability and an affordable price, can convert trimethylamine (TMA, a kind of degradation product) to trimethylamine-N-oxide (TMAO, amine oxide in living fishes) [11,12]. If the amount of TMAO is increased by H_2_O_2_ treatment, the protein of muscle tissue will be stabilized [13]. Moreover, the decrease of main glycoproteins on the fish skin will reduce viscosity and slow down the appearance of off-flavor [14]. Consuming these foods containing excessive H_2_O_2_ can cause nausea, headaches and potential risks of cancer [15,16,17]. The residual H_2_O_2_ must be removed from dairy foods during the processing of foods, according to US FDA regulations [18]. Consequently, a rapid and quantitative method of detecting H_2_O_2_ residue in fishery products is sorely needed to guarantee food safety.

Among a large number of the H_2_O_2_ physicochemical-sensing strategies, including chemiluminescence [19], titrimetry [20], spectrophotometry [21], electrochemistry [22], etc., the electrochemical sensor has become an optimal choice to actualize the H_2_O_2_ detection due to its high reliability, selectivity, low detection limits, simplicity of the device, and easy application in situ [23]. During the past several decades, although electrochemical enzyme-based sensors have attracted strong interest and developed extensively [24,25], the sensing mechanism that is based on biorecognition elements makes the enzyme sensors lack reproducibility and long-term stability. The denaturation process may even be accelerated in some special environments [26]. In consequence, developing advanced nonenzymatic sensing materials with enzyme–mimetic catalytic activity to improve the performance of the electrodes for H_2_O_2_ detection has attracted increasing attention, including transition metals [27], noble metals [28], metal oxides [29] and carbon-based materials [30].

Nanozymes are artificial nanomaterials with inherent catalytic properties similar to natural enzymes [31]. Recently, metal–organic frameworks (MOFs) compounds composed of metal–oxygen clusters bridged by organic linking molecules are becoming a new class of enzyme mimics [32]. The porous 3D coordination polymers have larger specific surface areas and a higher density of accessible catalytic sites [33], which makes it possible to be a type of excellent peroxidase mimicking materials [34,35,36]. It has been reported that MOFs constructed by Zr_6_ and porphyrin possess framework hyperstability for the strong electrostatic interactions between high valence Zr^IV^ and carboxylate linkers [37,38]. Among the reported porphyrinic Zr-MOFs, due to their nanoporous channels and extraordinary chemical stability, PCN-224 (PCN = porous coordination network) is considered an impressive material for practical applications in aqueous media [39,40], which paves the way for practical applications on electrodes.

Fe^II^ and Fe^III^ are common and easily accessible redox states, which make iron a good candidate in preparing Fe-based MOFs [41,42]. Inspired by the peroxidase-like activity of natural metalloporphyrins, such as heme, iron porphyrin also has been used as MOF materials to model peroxidase [43,44]. The reaction of H_2_O_2_ and iron ions can provide a basis for Fe-based MOFs as H_2_O_2_ nanozymes [45,46]. Therefore, we implanted coordinatively unsaturated Fe^III^ ions into the porphyrin unit in PCN-224 and generated a new hybrid structure, Fe@PCN-224, by merging the above advantages of PCN-224 and Fe^III^ [47]. In the kinetics study with three typical peroxidase substrates (3,3′,5,5′-tetramethylbenzidine, 2,2′-azinodi(3-ethylbenzothiazoline)-6-sulfonate, and o-phenylene-diamine), the newly formed Fe@PCN-224 possesses peroxidase-like activity with much lower Michaelis constants (K_m_) and higher K_cat_ values (maximal reaction velocity divided by catalyst molar concentration, viewed as the optimum turnover rate demonstrating the catalytic activity) than most of the peroxidase-like nanozymes, indicating their enhanced catalytic activities [48].

In this study, a new H_2_O_2_ electrode was developed by coating Fe@PCN-224/Nafion on the glassy carbon electrode (GCE). As dispersant and interferent barrier, Nafion formed uniform membranes that immobilized Fe@PCN-224 on the GCE surface. Scanning electron microscopy (SEM) was adopted to characterize the morphology of the composite materials. Cyclic voltammetry (CV) was used to examine the performance of the sensor. Furthermore, stability, selectivity, reproducibility, linear range and detection limit were proposed and discussed. In this work, as-prepared Fe@PCN-224/Nafion/GCE was employed to detect residual H_2_O_2_ in three kinds of fresh fishery products. The relative accuracy assessment of the electrochemical method was also attempted by comparing it with the measurement results of the photometric method.

## 2. Materials and Methods

### 2.1. Materials

Hydrogen peroxide (H_2_O_2_, 30%), horseradish peroxidase (HRP, freeze-dried powder, >200 units/mg), N,N-diethyl-1,4-phenylenediammonium sulfate (DPD), L-tyrosine and L-phenylalanine were purchased from Aladdin (Shanghai, China). FeCl_3_ and Nafion (10% in water) were purchased from Sigma-Aldrich (Shanghai, China). Tetrakis(4-carboxyphenyl)porphyrin (H_2_TCPP) was purchased from TCI (Shanghai, China). Oxalic acid dehydrate was purchased from Mackin (Shanghai, China). ZrClO_2_·8H_2_O, benzoic acid, DMF, glucose, ascorbic acid, potassium dihydrogen phosphate, lithium carbonate, sodium phosphate monobasic dihydrate, sodium phosphate dibasic dodecahydrate, magnesium sulfate heptahydrate, and calcium chloride dehydrate were purchased from Sinopharm Chemical Reagent Co., Ltd. (Shanghai, China). All the experimental water comes from the Milli-Q reference system (Millipore, America) unless stated otherwise.

Fishery product samples were *Todarodes pacificus* belonging to the family Ommastrephidae; *Larimichthys polyactis* belonging to the family Sciaenidae; *Pennahia argentata* belonging to the family Sciaenidae. The above three kinds of fresh fishery products were purchased in a local market (Xincheng Market, No. 95 Jindao Road, Dinghai District, Zhoushan 316021, China) and brought into the laboratory no later than 1 h in ice.

### 2.2. Instruments

A scanning electron microscope (Zeiss Sigma 500, Oberkochen, Germany) was used to analyze morphology. A UV-visible spectrophotometer (Evolution 300, Waltham, MA, USA) was applied to detect H_2_O_2_ concentration. Cyclic voltammetry (CV) and amperometric measurements were performed using a PARSTAT 4000 electrochemical workstation (AMETEK, Princeton, NJ, USA). All electrochemical measurements were carried out in a typical three-electrode system with saturated calomel electrode (SCE) as the reference electrode, platinum (Pt) disk electrode as the counter electrode and modified glassy carbon electrode (GCE) as the working electrode.

### 2.3. Fabrication of PCN-224 and Fe@PCN-224

PCN-224 nanoparticles were manufactured according to the previously reported procedure [48]. In this experiment, 50 mg of H_2_TCPP, 150 mg of ZrOCl_2_·8H_2_O, and 1.4 g of benzoic acid were first dissolved in 50 mL of DMF. The solution was heated evenly at 90 °C for 5 h. After the reaction was completed, PCN-224 nanoparticles were collected by centrifugation and then washed three times with fresh DMF.

As for Fe@PCN-224, 60 mg of PCN-224 and 80 mg of FeCl_3_ were dispersed in 20 mL DMF. The solution was stirred for 30 min at room temperature and then heated at 120 °C under stirring (300 rpm) for 8 h. Finally, the Fe@PCN-224 were obtained by centrifugation and washed three times with DMF and were stored in fresh DMF for further analysis.

### 2.4. Preparation of Fe@PCN-224-Modified Electrodes

The above Fe@PCN-224 was washed three times with water and 100 μL 1wt% Nafion were mixed under ultrasonication for 1 h, so the Fe@PCN-224/Nafion composites were obtained. Before fabrication of the electrodes, the treatments of bare glassy carbon electrodes were required. Glassy carbon electrode was first polished with 1.5 μm, 0.5 μm, and 50 nm alumina slurries to create a mirror finish, and then sonicated with ultra-pure water, 1:1 nitric acid, ethanol, and ultra-pure water successively. After the glassy carbon electrode was dried by nitrogen gas, 5 μL of Fe@PCN-224/Nafion suspension was coated on the glassy carbon electrode surface, forming Fe@PCN-224/Nafion/GCE upon drying overnight under room temperature.

### 2.5. Sample Treatment for H_2_O_2_ Determination

Preparation of fresh fishery products: 2 g muscle samples of the fresh fishery products were chopped and extracted with 30 mL 0.1 M phosphate buffer. The samples were obtained by centrifugation (6000 rpm, 15 min) and filtration with a 0.45 µm filter membrane.

Preparation of some samples with H_2_O_2_ to simulate illegal treatment: 2 g muscle samples of the fresh fishery products were completely immersed in 10 mL H_2_O_2_ solution (0.8%) for 2 min [7]. The samples were rinsed 3 times with 50 mL of fresh water after removal of the liquid. The same method as above was used to extract the treated fishery products.

### 2.6. Electrochemical Determination of H_2_O_2_

All electrochemical measurements were carried out in a three-electrode electrochemical cell at room temperature of 25 °C. CVs were obtained with a potential window of −1.5–1.5 V at a scan rate of 50 mV/s in 0.1 M phosphate buffer (pH 7.0). All amperometric measurements were carried out at an applied potential of 1.0 V in 0.1 M phosphate buffer (pH 7.0) without specific description, requiring the transient background to decrease to a steady-state value. Magnetic stirring was applied to the solution during amperometric measurements to maintain convective mass transfer characteristics.

### 2.7. Spectrophotometric Determination of H_2_O_2_

We used the photometrical method described by Bader et al. [49], which was slightly modified by Drabkova et al. [50]. The buffer stock solution was prepared by mixing 1.2 mL 0.5 M phosphate buffer (pH 7.0) and 10.8 mL sample. 20 μL of DPD reagent (0.1 g DPD diluted in 10 mL 0.5 M H_2_SO_4_ solution) and 20 μL of HRP reagent (10 mg HRP diluted in 10 mL water) were added into the buffered sample, while continually stirred. The developed color was measured at a wavelength of 551 nm. The absorbance of the whole mixture without HRP addition was measured as a blank. The concentration of hydrogen peroxide was calculated according to the following equation:

(1) $${\left[H_{2}O_{2}\right]}_{\mathrm{sample}}=\frac{∆A^{551}V_{\mathrm{final}}}{{\varepsilon \mathrm{lV}}_{\mathrm{sample}}}$$

in which ΔA^551^ is absorbance after subtracting the value of the blank, *V*_final_ is the final volume of the measured mixture, *ε* is 21,000 M^−1^ cm^−1^, *L* is the length of the optical cell, and *V*_sample_ is the volume of the original sample.

## 3. Results and Discussion

### 3.1. Characterization of Composites

**Figure 1:**
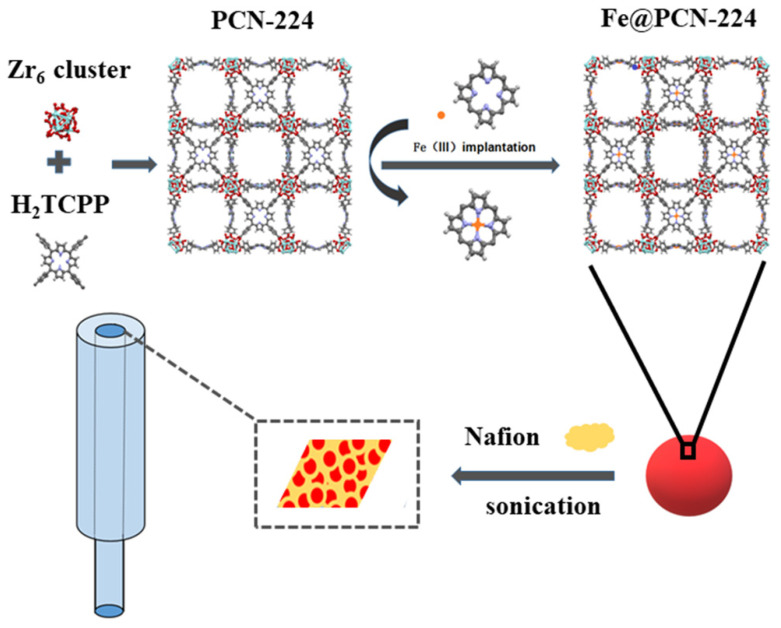
Schematic illustration for the fabrication of Fe@PCN-224/Nafion/glassy carbon electrode (GCE).

The preparation of Fe@PCN-224/Nafion/GCE was a four-step process, as presented in Figure 1. First, prior to the preparation of Fe@PCN-224, PCN-224 was synthesized using typical methods [48]. Due to the strong interaction between Zr_6_ and the carboxyl group, PCN-224 has super stability. Second, free Fe^III^ ions were incorporated into the center of the porphyrin unit. The obtained Fe@PCN-224 owns high open metal site density for electrochemical applications due to the special structure of MOFs [51]. Then, by mixing the Fe@PCN-224 particles in Nafion solution in a fast ultrasonic process, the well-mixed Fe@PCN-224/Nafion composites were obtained. Nafion, as a proton-conducting membrane in electrochemical sensors, has the ability to block the anionic oxidant and reductant, which is expected to avoid interference in real samples [52]. Finally, Fe@PCN-224/Nafion suspension was coated on the GCE surface, forming Fe@PCN-224/Nafion/GCE upon drying. Surface structure and response properties were obtained by subsequent characterization analysis and electrochemical experiments.

**Figure 2:**
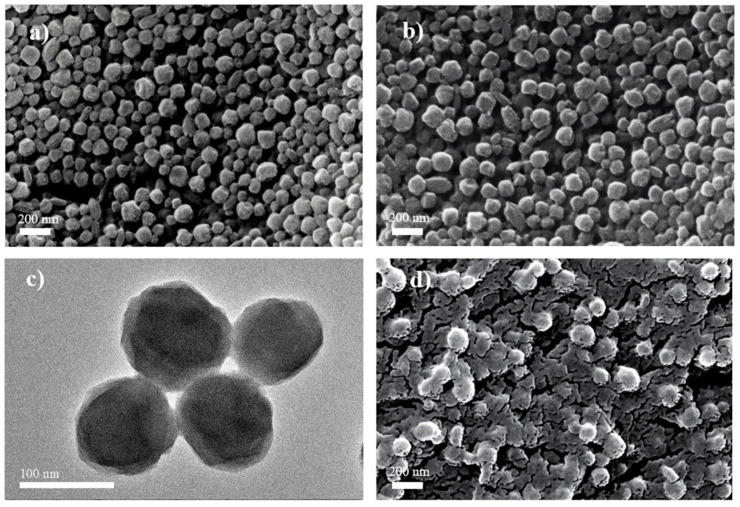
SEM images of (**a**) PCN-224, (**b**) Fe@PCN-224, (**d**) Fe@PCN-224/Nafion. TEM image of (**c**) Fe@PCN-224.

The texture of PCN-224 (a), Fe@PCN-224 (b, c) and Fe@PCN-224/Nafion (d) can be observed in SEM and TEM images presented in Figure 2. SEM image shows that PCN-224 is a uniform spherical particle with the size of approximately 100 nm (Figure 2a), which is beneficial for evenly mixing. Fe@PCN-224 displays almost the same morphology and size as PCN-224 after the reaction between Fe^III^ and PCN-224 (Figure 2b,c). Nafion is seen as a dense sheet of planes with wrinkles stacked on it. From the SEM image of Fe@PCN-224/Nafion (Figure 2d), Nafion sheets form a stratified structure by intersecting together on the surface of Fe@PCN-224. The interface between the Nafion sheets and Fe@PCN-224 is coherent, enabling efficient electron transfer within the hybrid structure. Figure S1 shows corresponding elemental mapping images of Fe@PCN-224/Nafion. Through element map identification, Fe^III^ was successfully incorporated into PCN-224, and the Fe@PCN-224 was evenly mixed with Nafion.

### 3.2. Cyclic Voltammetry of the H_2_O_2_ Sensor

**Figure 3:**
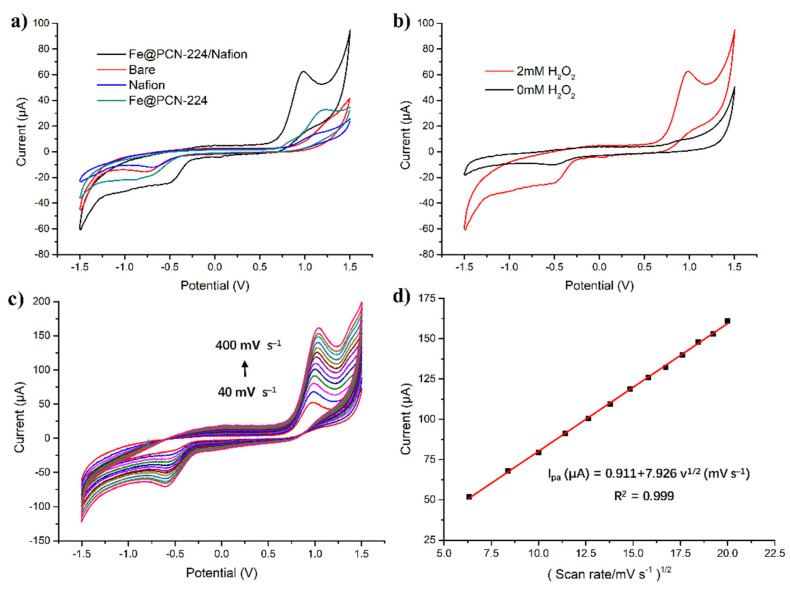
Cyclic voltammograms of (**a**) the Fe@PCN-224/Nafion/GCE, bare, Nafion, and Fe@PCN-224-modified GCE with 2 mM H_2_O_2_ and (**b**) Fe@PCN-224/Nafion/GCE with (red curves) or without (black curves) 2 mM H_2_O_2_ in pH 7.0 phosphate buffer recorded at scan rate of 50 mV s^−1^, respectively. (**c**) Cyclic voltammograms of Fe@PCN-224/Nafion/GCE for 2 mM H_2_O_2_ in different scan rates (40, 70, 100, 130, 160, 190, 220, 250, 280, 310, 340, 370 and 400 mV s^−1^). (**d**) The relationship between peak currents and scan rates.

The sensing properties of Fe@PCN-224/Nafion/GCE for the electrochemical detection of H_2_O_2_ were studied preliminarily using cyclic voltammetry (CV). Figure 3a shows the CVs of the bare GCE, the Nafion/GCE, the Fe@PCN-224/GCE and the Fe@PCN-224/Nafion/GCE with 2 mM H_2_O_2_ at a scan rate of 50 mV s^−1^. For the bare GCE and Nafion-coated electrodes, a very weak oxidation peak at about 1.2 V is observed, which suggests that GCE and Nafion have no catalytic effects on the reaction of H_2_O_2_. A similar phenomenon was observed in the H_2_O_2_ sensor (Cu-TDPAT/GCE) developed by Zhang et al. [53], which was attributed to the slow electron transfer kinetics of the H_2_O_2_ oxidation process. In contrast, CVs of Fe@PCN-224/GCE and the Fe@PCN-224/Nafion/GCE exhibit a remarkable oxidation current peak at about 1.1 V, which are approximately 4.7 and 2.5 times higher than the Nafion/GCE, respectively, indicating that Fe@PCN-224 has an efficient electrocatalytic activity for H_2_O_2_ oxidation. Comparing the CVs of Fe@PCN-224/Nafion/GCE and Fe@PCN-224/GCE, the oxidation current of Fe@PCN-224/Nafion/GCE is almost 1.9 times higher than that of Fe@PCN-224/GCE, which is rational because of better conductivity caused by Nafion. Moreover, the synergistic effect of Nafion and Fe@PCN-224 could result in an amplified oxidation current. The CV of Fe@PCN-224/Nafion/GCE in blank solution, shown in Figure 3b, exhibits one pair of redox peaks with extremely weak oxidation currents. After adding 2 mM H_2_O_2_ in solution, a pair of enhanced redox peaks are observed. The oxidation peak current increases 6.8 times higher than in the blank solution, demonstrating appreciable electrocatalytic activity of the Fe@PCN-224/Nafion/GCE toward H_2_O_2_ oxidation.

Fe@PCN-224 shows nearly no emission peak by TA (terephthalic acid) probing method, which demonstrates that Fe@PCN-224 does not produce ^•^OH [48]. TA could react with ^•^OH to form TA-OH, which is fluorescent at an excitation wavelength of 315 nm [54,55]. Some researchers suggest that the Fenton reaction produces not only ^•^OH but also the ferryl ion (Fe^4+^=O), which is dependent on the nature of the chelator [56]. Fe@PCN-224/Nafion probably produces ferryl ion in the presence of H_2_O_2_ to exhibit peroxidase-like activity [48,57,58], which needs further studies to demonstrate. Based on the above results, the pertinent reaction mechanism could be proposed as two procedures: In the first step, the catalytic center Fe^III^@PCN-224/Nafion is oxidized electrochemically to Fe^IV^=O@PCN-224/Nafion. The second procedure is the progress of chemical recognition. H_2_O_2_ can be absorbed to the pores and surfaces of Fe^IV^=O@PCN-224/Nafion, then Fe^IV^=O@PCN-224/Nafion reacts simultaneously with H_2_O_2_ and is reduced to Fe^III^@PCN-224/Nafion. H_2_O_2_ loses electrons and is oxidized to produce oxygen. The reaction mechanism could be described as follows:

(2) $${\mathrm{Fe}}^{\mathrm{III}}@\mathrm{PCN}-224/\mathrm{Nafion}+H_{2}O\to {\mathrm{Fe}}^{\mathrm{IV}}=O@\mathrm{PCN}-224/\mathrm{Nafion}+2H^{+}+e^{-}$$

(3) $$2{\mathrm{Fe}}^{\mathrm{IV}}=O@\mathrm{PCN}-224/\mathrm{Nafion}+H_{2}O_{2}+2H^{+}\to 2{\mathrm{Fe}}^{\mathrm{III}}@\mathrm{PCN}-224/\mathrm{Nafion}+2H_{2}O+O_{2}↑$$

The effect of the scan rate versus the current in 2 mM H_2_O_2_ solution was detected. Referring to Figure 3c, the oxidation peak current (I_pa_) increases with the scan rate (v) in the range of 40 to 400 mV s^−1^. There is a good linear relationship between I_pa_ and the square root of v with *R*^2^ = 0.999 (Figure 3d). The relationship could be expressed as I_pa_ (μA) = 0.911+7.926v^1/2^ (mV s^−1^), which indicates that the electrochemical reaction of H_2_O_2_ on Fe@PCN-224/Nafion/GCE is a diffusion-controlled irreversible process in the investigated potential range.

### 3.3. Amperometric Measurement of H_2_O_2_

**Figure 4:**
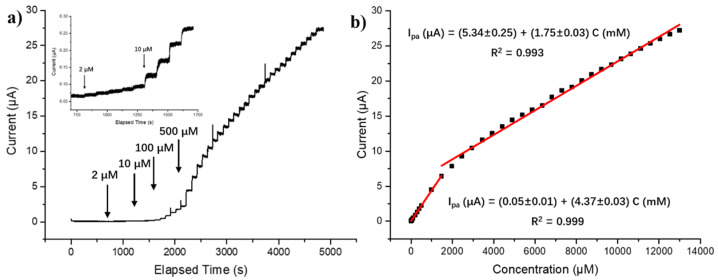
(**a**) The typical current–time dynamic response of the Fe@PCN-224/Nafion/GCE with successive additions of H_2_O_2_ ranging from 2–13,000 μM. Inset: enlarged current–time response curve with H_2_O_2_ concentrations ranging from 2–50 μM. (**b**) The linear relationship between current signal and H_2_O_2_ concentration ranging from 2–1500 μM and 1500–13,000 μM.

As shown in Figure S2, the oxidation current response of the sensor gradually increases with the increase of applied potential (0.8–1.0 V) and decreases after this (1.0–1.2 V), reaching a maximum value at 1.0 V. Therefore, 1.0 V was selected as the optimal applied potential in subsequent measurements. Typical current–time dynamic response of the Fe@PCN-224/Nafion/GCE towards H_2_O_2_ is shown in Figure 4a. The electrode responds quickly to the change of H_2_O_2_ concentration. The current is stable within 10 s after adding different concentrations of H_2_O_2_. The linear plot of H_2_O_2_ concentration versus amperometric currents demonstrates two corresponding linear regions of 2 to 1500 μM and 1500 to 13,000 μM, which covers four orders of magnitude of H_2_O_2_ concentrations. As illustrated in Figure 4b, the corresponding calibration curve in range from 2 to 1500 μM exhibits regression equation I_pa_ (μA) = (0.05 ± 0.01) + (4.37 ± 0.03) C (mM), *R*^2^ = 0.999. In range from 1500 to 13,000 μM, the corresponding calibration curve could be expressed as regression equation I_pa_ (μA) = (5.34 ± 0.25) + (1.75 ± 0.03) C (mM), *R*^2^ = 0.993. The detection limit is 0.7 μM with a signal-to-noise ratio of three (S/N = 3).

A comparison of linear range and detection limit for Fe@PCN-224/Nafion/GCE with other H_2_O_2_ sensors reported in the literature is shown in Table 1. The proposed electrode has a wider range than traditional sensors, especially some horseradish peroxidase sensors. The wider linear range, including four orders of magnitude, allows the electrode to monitor a broader range of H_2_O_2_ concentrations. It can be seen that Fe@PCN-224/Nafion/GCE is able to present satisfactory sensing performance with a wide linear range and a comparable detection limit.

The good performance of the Fe@PCN-224/Nafion/GCE may be attributed to two main reasons. First of all, Nafion can block the anionic oxidant and reductant, which helps to attenuate their interference and extend the service life of the H_2_O_2_ sensor. Second, Fe@PCN-224 is highly porous and provides a microenvironment for H_2_O_2_ in the pores, where Fe@PCN-224 furnishes plenty of open metal active sites of Fe^III^. The open metal active sites of Fe^III^ exhibit enzyme-like activity with H_2_O_2_ and play a part as the catalytic center.

However, fluctuation could be observed during the detection process, which influences the detection limit of the electrode. The possible reason for the fluctuation of Fe@PCN-224/Nafion/GCE could be related to the relatively poor conductivity of the material. On one hand, the intrinsic insulating characteristics of the carboxyl groups utilized to form MOFs results in a low electrical conductivity, and electrons are obstructed from migrating along or accessing the skeleton of MOFs material. On the other hand, MOFs with micro size often have poor contact with the smooth surface of the electrode, making it difficult to transfer interfacial electrons from MOFs to the electrode surface.

### 3.4. Selectivity, Stability, and Reproducibility

**Figure 5:**
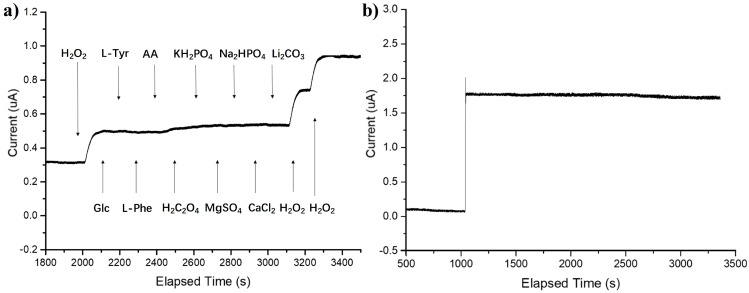
(**a**) Effects of ten interfering species on the response curve of Fe@PCN-224/Nafion/GCE in the presence of H_2_O_2_. Each arrow represents the addition of the corresponding substance at a concentration of 100 μm. (**b**) The dynamic response curve of Fe@PCN-224/Nafion/GCE towards 400 μM H_2_O_2_ over 2300 consecutive seconds.

Selectivity. Investigations of the selectivity of the Fe@PCN-224/Nafion/GCE to potential interferents were essential for practical applications. The public interferents of common H_2_O_2_ electrodes were chosen. Biological samples often contain electroactive reducing agents, which produce corresponding oxidation currents during the detection of H_2_O_2_ and seriously interfere with the determination. Figure 5a shows the response curve of Fe@PCN-224/Nafion/GCE to H_2_O_2_, Glc (glucose), L-Tyr (L-tyrosine), L-Phe (L-Phenylalanine), AA (Ascorbic Acid), H_2_C_2_O_4_, KH_2_PO_4_, MgSO_4_, Na_2_HPO_4_, CaCl_2_ and Li_2_CO_3_. After adding 100 μM H_2_O_2_, an obvious current response could be observed. The current does not change significantly with the subsequent addition of ten 100 μM interfering species, indicating a good selectivity for H_2_O_2_ sensing. Therefore, the high selectivity of the sensor makes it a potential candidate for H_2_O_2_ determination in complex media.

Stability. The current–time curve was continuously recorded to examine the stability of the modified electrode. As shown in Figure 5b, the current signal nearly remains unchanged for a long period of 2300 s, which suggests good stability of the sensor. Long-term measurement was also performed to confirm electrode stability. After storing the electrode in the air for 30 days, the current response decreases to 96.6% of the original response, indicating its good long-term stability.

Reproducibility. The reproducibility of the Fe@PCN-224/Nafion/GCE sensors prepared in different batches was also explored. Within seven Fe@PCN-224/Nafion/GCE sensors prepared, all of them can give a stable response in 100 μM H_2_O_2_ solution. Five sensors exhibit an average of 0.49 μA response current with a relative standard deviation (RSD) of 3.83%. However, two sensors exhibit a much lower measuring current compared to the other five, which may be related to the base GCE electrode. Further research should be carried out to find it.

### 3.5. Application of the H_2_O_2_ Sensor in Real Samples

As a cheap and effective preservative and bleaching agent, H_2_O_2_ is generally used excessively by some illegal vendors. Superfluous H_2_O_2_ residues in seafood and other foods pose a huge threat to consumer health. In order to testify the feasibility of the proposed Fe@PCN-224/Nafion/GCE for practical analysis, it was used to measure the accuracy of H_2_O_2_ concentration in *Todarodes pacificus*, *Larimichthys polyactis* and *Pennahia argentata*. We added the above three kinds of chopped fresh fishery products samples to the buffer and filtered them, then used the standard addition method to make calibration curves of H_2_O_2_ concentrations and amperometric currents, respectively. Figure S3 was the typical current–time dynamic curves and linear relationships, suggesting that Fe@PCN-224/Nafion/GCE has a good linear relationship with H_2_O_2_ in the range of 10–1500 μM in different fishery products samples. Then the concentration of H_2_O_2_ in illegally treated fishery products samples could be calculated by substituting the measured current values into the standard curves. A photometrical method was also applied to measure the H_2_O_2_ concentrations (considered as true values), which could help to assure the accuracy of H_2_O_2_ quantitative detection by the sensor. As shown in Table 2, the H_2_O_2_ concentrations detected by the electrochemical method are about 7% lower than those detected by the photometrical method. The difference between the two methods may be ascribed to the applied constant voltage in the electrochemical detection process. H_2_O_2_ could react with fishery products samples to bleach and prevent corrosion, thereby reducing the concentration of H_2_O_2_ remaining in the solution. During the electrode measurement, the applied constant voltage may accelerate the reaction of residual H_2_O_2_ and samples, which results in a decrease in H_2_O_2_ concentration. Consequently, the measured concentration by the electrochemical method is lower than that measured by the photometrical method.

In general, the accuracies of different fishery product samples are between 91% and 95% in Table 2. The comparable deviations indicate that this electrode could effectively detect H_2_O_2_ and resist the interference in real sample analysis. In addition to the advantage of fast and facile H_2_O_2_ detection, other chemical reagents are not required for using the prepared nanohybrid-modified electrode. Therefore, Fe@PCN-224/Nafion/GCE is expected to be used in real sample research such as seafood.

## 4. Conclusions

In summary, Fe@PCN-224 with excellent performance was successfully fabricated by incorporating FeIII into the center of PCN-224, and it was applied to fabricate a novel electrochemical sensor for the determination of H_2_O_2_ concentration. The sensor shows a high electrocatalytic ability to H_2_O_2_ oxidation in a wide linear range and exhibits outstanding anti-interference ability, splendid stability. The prepared nanohybrid-modified electrode can be used to determine H_2_O_2_ concentration in three kinds of fresh fishery products samples. In addition to the advantage of rapidness and briefness, simplicity of the device and easy application of Fe@PCN-224/Nafion/GCE open up new opportunities for in situ H_2_O_2_ detection in foods.

## Figures and Tables

**Table 1:** Comparison of different sensors for the determination of H_2_O_2._

Electrode Material	Linear Range (μM)	Detection Limit (μM)	Reference
^1^ MP/ZnO/PGE	1–100	0.3	[59]
^2^ HRP/SPE	5.98–35.36	0.48	[60]
^3^ Ag/L-Cys/GCE	2.5–1500	0.7	[28]
^4^ Cyt c/MPCE	20–240	14.6	[61]
^5^ C_12_-PPy-Au-HRP/GCE	2–420	0.25	[62]
Cu_2_O/^6^ GNs/GCE	300–7800	20.8	[63]
Nafion/^7^ Mb/CGNs/GCE	1.5–90	0.5	[64]
^8^ NG/Ag NP/MME	5–47,000	0.56	[65]
Fe@PCN-224/Nafion/GCE	2–13,000	0.7	this work

^1^ MP/ZnO/PGE = microperoxidase/zinc oxide/pyrolytic graphite electrode; ^2^ HRP/SPE = horseradish peroxidase/screen-printed electrode; ^3^ Ag/L-Cys = leaf-like silver/L-cysteine; ^4^ Cyt c/MPCE = cytochrome c/macroporous active carbon electrode; ^5^ C_12_-PPy = 1-dodecyl-3-methylimidazolium-polypyrrole; ^6^ GNs = graphene nanosheets; ^7^ Mb/CGNs = myoglobin/colloidal gold nanoparticles; ^8^ NG/Ag NP/MME = nanoscale graphene/Silver nanoparticles/membrane-modified electrode.

**Table 2:** H_2_O_2_ concentration detected in fresh fish samples by Fe@PCN-224/Nafion/GCE and Photometrical method.

Samples	Fe@PCN-224/Nafion/$ GCE (µmol kg^−1^)	Photometrical Method (µmol kg^−1^)	Accuracy (%)
Todarodes pacificus	18.1 ± 0.2	19.9 ± 0.2	91.0
Larimichthys polyactis	0.71 ± 0.08	0.76 ± 0.09	94.1
Pennahia argentata	2.00 ± 0.03	2.13 ± 0.05	93.6

Accuracy (%) means the ratio of H_2_O_2_ concentrations detected by the Fe@PCN-224/Nafion/GCE to; H_2_O_2_ concentrations detected by the photometrical method.

## Data Availability

The data presented in this study are available in this article and supplementary material.

## Supplementary Materials

The following are available online at https://www.mdpi.com/2304-8158/10/2/419/s1, Figure S1: SEM image of Fe@PCN-224/Nafion and its corresponding elemental mapping images, Figure S2: Effects of applied potential in 0.1 M phosphate buffer (pH 7.0) containing 2 mM H_2_O_2_ on the current responses of Fe@PCN-224/Nafion/GCE, Figure S3: The typical current–time dynamic response of the Fe@PCN-224/Nafion/GCE and the linear relationship in *Todarodes pacificus*, *Larimichthys polyactis* and *Pennahia argentata*.
