# Supplementary material for: A Long-Term Stable Sensor Based on Fe@PCN-224 for Rapid and Quantitative Detection of H2O2 in Fishery Products
Source: Foods. 2021 Feb 14;10(2):419. doi: 10.3390/foods10020419 (PMC7918592; doi:10.3390/foods10020419)
Supplement: Supplementary file 1 [file foods-10-00419-s001.pdf]

Supplementary Materials

A long-term stable sensor based on Fe@PCN-224 for rapid and quantitative detection of H_2O_2 in fishery products

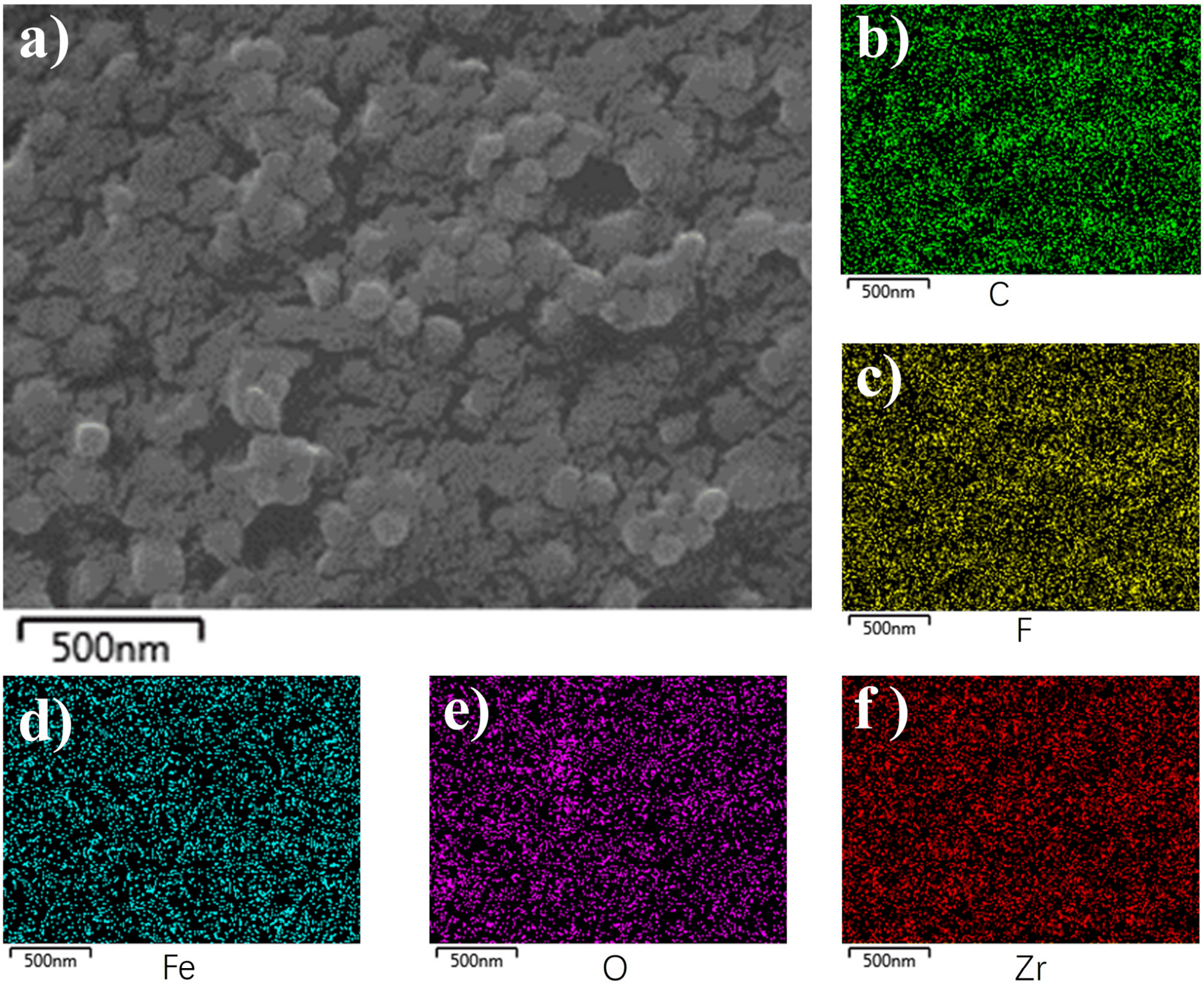

Fig. S1. SEM image of Fe@PCN-224/Nafion (a) and its corresponding elemental mapping images, (b) C, (c) F, (d) Fe, (e) O, (f) Zr.

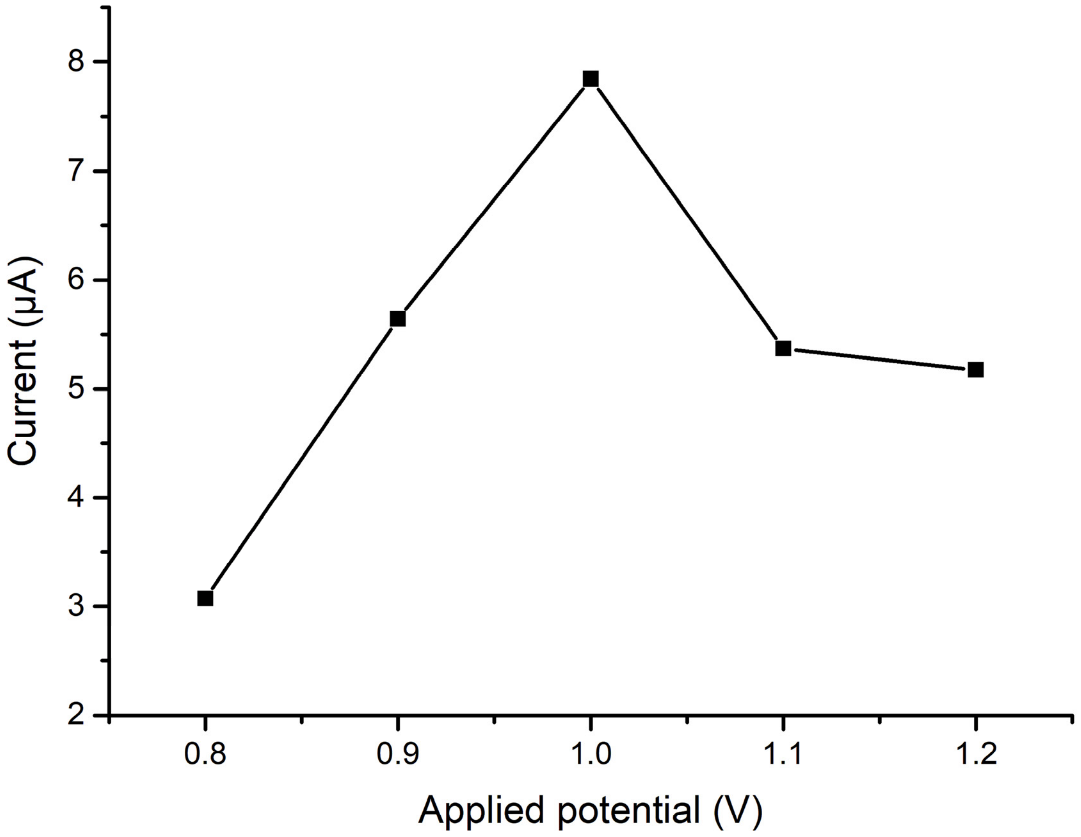

Fig. S2. Effects of applied potential in 0.1 M phosphate buffer (pH 7.0) containing 2 mM H_2O_2 on the current responses of Fe@PCN-224/Nafion/GCE.

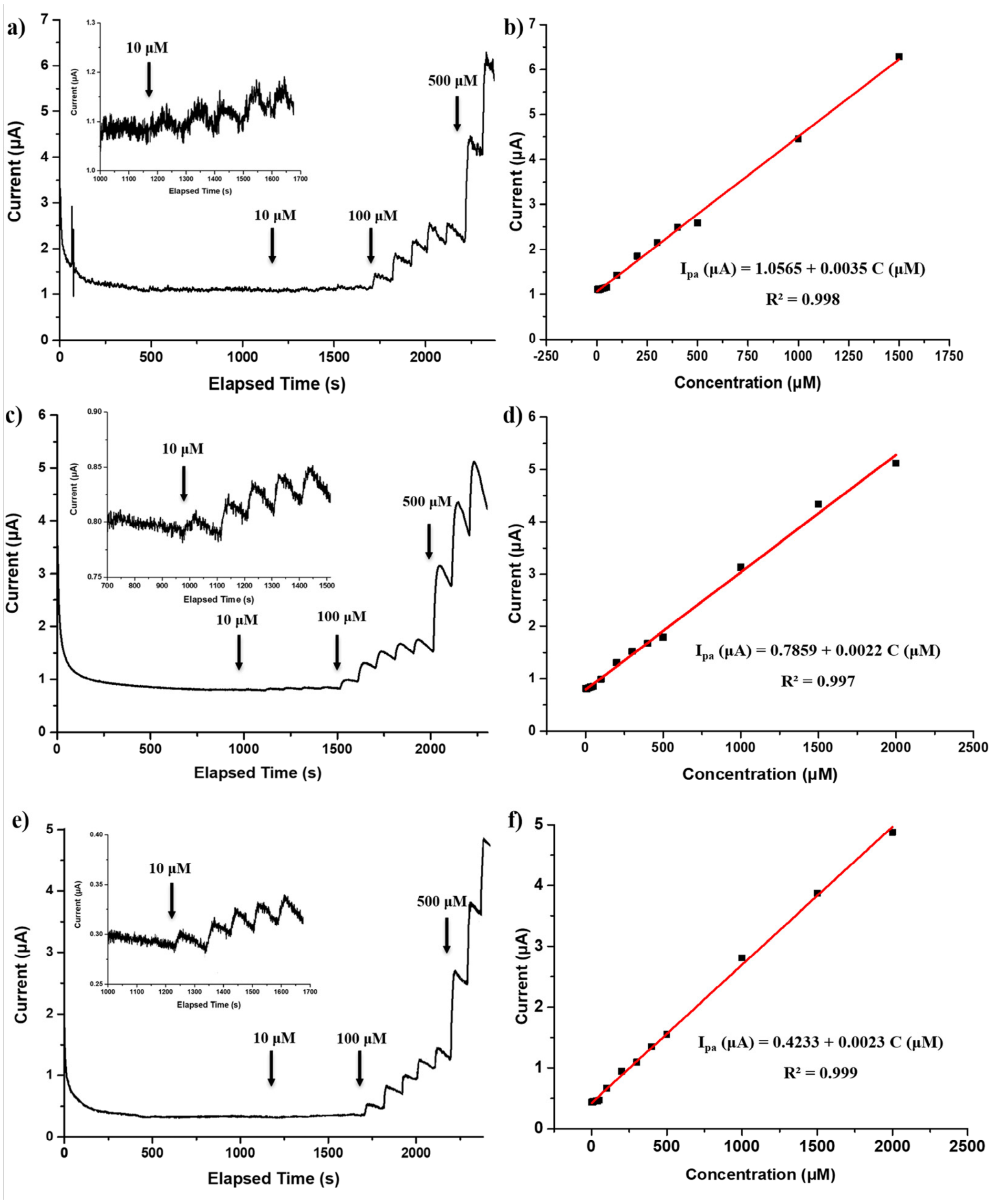

Fig. S3. The typical current–time dynamic response of the Fe@PCN-224/Nafion/GCE with successive additions of H_2O_2 in (a) Todarodes pacificus, (c) Larimichthys polyactis and (e) Pennahia argentata. Inset: Enlarged current–time response curve with H_2O_2 concentrations. The linear relationship between current signal and H_2O_2 concentration in (b) Todarodes pacificus, (d) Larimichthys polyactis and (f) Pennahia argentata.
